# Broadband achromatic metalens for high-resolution imaging

**DOI:** 10.1038/s41377-025-01858-2

**Published:** 2025-05-22

**Authors:** Yangkyu Kim, Inki Kim

**Affiliations:** 1 Department of Biophysics, Institute of Quantum Biophysics, Sungkyunkwan University, Suwon, 16419 Republic of Korea; 2 Department of Intelligent Precision Healthcare Convergence, Sungkyunkwan University, Suwon, 16419 Republic of Korea; 3 Department of MetaBioHealth, Sungkyunkwan University, Suwon, 16419 Republic of Korea

**Keywords:** Metamaterials, Imaging and sensing

## Abstract

Introduction of the stepwise phase dispersion compensation layer allowed broadband achromatic metalens to have a high numerical aperture, which enabled high-resolution metalens imaging.

## Abstract (web summary)

A broadband achromatic metalens with high NA is demonstrated by a concept of stepwise phase dispersion compensation.

## Graphical abstract

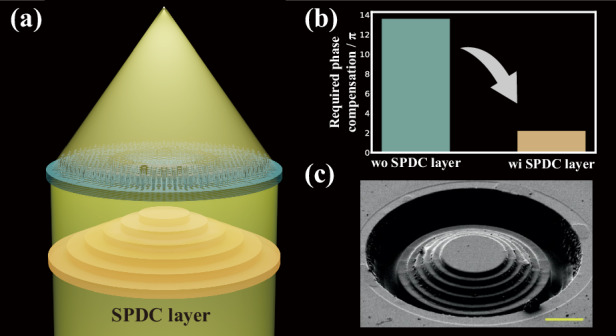

Metamaterials are artificial engineering structures designed in patterns by building blocks with subwavelength-sized nanostructures called meta-atoms, which can control various types of physical energy and are mainly used for light control^1^. A metasurface is a type of metamaterial that reduces the dimensionality of metamaterials and enables planar photonics by effectively controlling the phase of the incident light according to the arrangement of meta-atoms, which scatter light at the interface^1,2^. Metasurface-based lenses are called metalenses, and unlike conventional refractive lenses used in microscopes and cameras, they are diffraction-based optical elements. Compared to refractive lenses, metalenses are lighter and smaller in size. They can accurately implement lens phase profiles in nanometers through lithography without relying on molding, an old technology, positioning them as a suitable substitute for refractive lenses in the future. Therefore, related research is constantly being conducted to this day.

A metalens was manufactured based on plasmonic nanoslits^3^ or antenna^4^ using metals that can focus light to the subwavelength region and easily change the phase of light depending on the size and shape of the nanostructure. However, because the absorption loss of the metal reduces the efficiency of the lens, the metal was replaced with a dielectric material with relatively little absorption loss. Dielectric meta-atoms act like waveguides and achieve phase accumulation using the following equation^2^

$$\phi =\frac{\omega }{c}{n}_{{eff}}H$$

where ${n}_{{eff}}$ and $H$ denote the effective refractive index and height of the meta-atom, respectively, both of which are used to adjust the phase. It is advantageous for dielectric materials to have a high refractive index to obtain a wide phase difference. Thus, silicon^5,6^ (Si), gallium nitride^7^ (GaN), silicon nitride^8^ (Si_3_N_4_), and titanium dioxide^9^ (TiO_2_) are used as dielectric materials. In particular, GaN^10^, Si_3_N_4_^11^, and TiO_2_^12^, with high transmission in the incident light wavelength region, are preferred for imaging to increase focusing efficiency by minimizing light loss in the visible range.

However, these metalenses are unsuitable for general cameras or microscopes due to severe defects in the image obtained caused by chromatic aberration when the light source is a broadband multiwavelength. This chromatic aberration is due to dispersion by the difference in accumulated phases depending on the wavelengths during diffraction, and negative dispersion occurs when longer wavelengths are diffracted at a larger angle, contrary to dispersion by refraction^13,14^. To correct chromatic aberration, normal dispersion (or positive dispersion) by meta-atoms can be introduced to offset the negative dispersion, a process known as phase compensation^14^. Metalenses using GaN^15^, Si_3_N_4_^16^, and TiO_2_^17^ achieved imaging with chromatic aberration correction for the broadband wavelength through linear phase compensation. In addition, asymptotic phase compensation has led to the development of achromatic metalens that can cover a wider wavelength range (400–1000 nm)^13^.

Another challenge is developing achromatic metalens with high numerical aperture (NA) to increase image resolution. To have a broadband operating wavelength range and a high NA simultaneously, a very large phase dispersion value is required. However, it is impossible to compensate for this value using meta-atoms alone because the refractive index (2–3) and height (400–1000 nm) are physically and manufacturingly limited. To overcome this, RGB-achromatic metalenses^18,19^ focusing only on discrete multiwavelengths or multilayered metalenses^19,20^ were developed. However, developing broadband achromatic metalenses with high NA is still an ongoing task because the manufacturing process is complicated to commercialize, the image quality is poor, and wavelengths with chromatic aberrations still exist within the operating wavelength range.

In Light: Science & Applications, a newly published paper introduced broadband achromatic metalens with high NA developed by Jingen Lin and colleagues from the State Key Laboratory of Optoelectronic Materials and Technologies, School of Physics, Sun Yat-Sen University, Guangzhou, China^21^. By introducing stepwise phase dispersion compensation (SPDC), the authors developed a metalens with a maximum NA of 0.9 corrected for chromatic aberration in a 650–1000 nm bandwidth (Fig. 1a). The developed metalens showed a focusing efficiency of 22.4–29.1% in simulation against about 10% in experiments due to imperfections in the manufacturing process, especially the deviation in stair manufacturing. This metalens simultaneously has the highest NA, largest aperture size, and thin thickness among the achromatic metalenses developed. Its high NA was demonstrated through white light imaging with a high resolution of 1.10 µm.

**Fig. 1:**
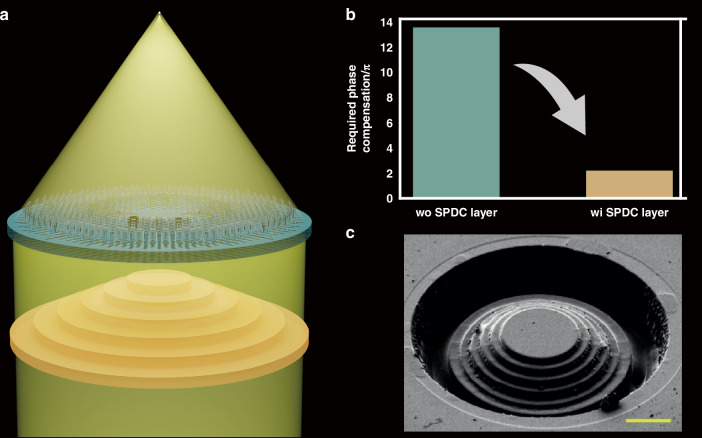
Broadband high NA achromatic metalens. **a** Schematic diagram of metalens with SPDC layer. **b** Phase compensation required for meta-atoms to correct chromatic aberration in high NA metalens. **c** The scanning electron microscope image of SPDC layer (Scale bar, 10 μm)^21^

This metalens consists of a total of five types of symmetrical crystalline silicon (c-Si) meta-atoms with a height of 500 nm and a period of 300 nm. The meta-atom arrangement was placed over an SPDC layer consisting of SiO_2_ and GaN. The SPDC layer, which consists of a total of six steps, made an optical path length difference according to the refractive index difference between SiO_2_ and GaN by varying the thickness of GaN according to the step and adjusting the phase. The SPDC layer reduced the very large phase dispersion value by the high NA and broadband wavelength range, which cannot be compensated for by meta-atom alone. The size and NA of the developed metalens required a meta-atom capable of up to 13.60π phase dispersion compensation. By introducing SPDC, chromatic aberration correction of the metalens was achieved with meta-atoms capable of phase compensation of up to 2.20π (Fig. 1b). The thickness and width of the SPDC layer were determined on the basis of the meta-atom that compensated for the maximum phase dispersion in the meta-atom library (Fig. 1c). Through this method, high NA values of 0.9 and 0.7 were achieved for two different broadband achromatic metalenses with radii of 20.1 $\mu m$ and 30.0 $\mu m$, respectively.

The results of these studies can be employed in various fields, such as cameras, microscopes, and near-eye displays. It can be developed suitably for light-field imaging by adjusting the field of view by configuring an achromatic metalens array^16,22^ or by expanding the lens size by introducing an SPDC layer with more steps. In addition, the developed metalens of high NA can provide bioimaging^23^ for observing cells and biomolecules in high resolution. Furthermore, it can effectively detect single molecules by enabling a small detection volume in fluorescence correlation spectroscopy^24^. Augmented reality (AR), virtual reality (VR)^18^, and holograms^25,26^, which were implemented with existing discrete operating wavelengths, can be expanded to a continuous broadband wavelength to enable the use of more colorful colors. Axial multifocal metal lenses enable three-dimensional imaging in biomedical imaging fields, and when combined with this technology, it allows high-resolution 3D imaging of organoids or cells by selecting various wavelengths^27^.
